# Enhanced YOLO11 for lightweight and accurate drone-based maritime search and rescue object detection

**DOI:** 10.1371/journal.pone.0321920

**Published:** 2025-07-01

**Authors:** Beigeng Zhao, Jiawen Zhao, Rui Song, Lizhi Yu, Xia Zhang, Jiren Liu

**Affiliations:** 1 School of Computer Science and Engineering, Northeastern University, Shenyang, Liaoning, China; 2 College of Public Security Information Technology and Intelligence, Criminal Investigation Police University of China, Shenyang, Liaoning, China; 3 Yuhong Sub-bureau, Shenyang Public Security Bureau, Shenyang, Liaoning, China; 4 Neusoft Corporation, Shenyang, Liaoning, China; University of Hradec Kralove: Univerzita Hradec Kralove, CZECHIA

## Abstract

Accurately and rapidly detecting objects and their locations in drone-captured images from maritime search and rescue scenarios provides valuable information for rescue operations. The YOLO series, known for its balance between lightweight architecture and high accuracy, has become a popular method among researchers in this field. Recent advancements in the newly released YOLO11 model have demonstrated significant progress in general object detection tasks across everyday scenarios. However, its application to the specific task of drone-based maritime search and rescue still leaves substantial room for improvement. To address this gap, we propose targeted optimizations to enhance YOLO11’s performance in this domain. These include integrating a Space-to-Depth module into the Backbone, incorporating a content-aware upsampling algorithm in the Neck, and adding an extra detection head to better exploit shallow image features. These modifications significantly improve the model’s ability to detect small, overlapping, and rarely occurring objects, which are common challenges in maritime search and rescue tasks. Experimental evaluations conducted on the large-scale SeaDronesSee dataset demonstrate that the proposed optimized YOLO11 outperforms YOLOv8, YOLO11, and MambaYOLO across all scales. Moreover, under lightweight configurations, the model achieves substantial performance gains over YoloOW, a method renowned for its accuracy but depends on heavyweight configurations. In the lightweight complexity range, the proposed model achieves a relative accuracy improvement of 20.85% to 43.70% compared to these state-of-the-art methods. The code supporting this research is available at https://github.com/bgno1/sds_yolo11.

## Introduction

**Fig 1:**
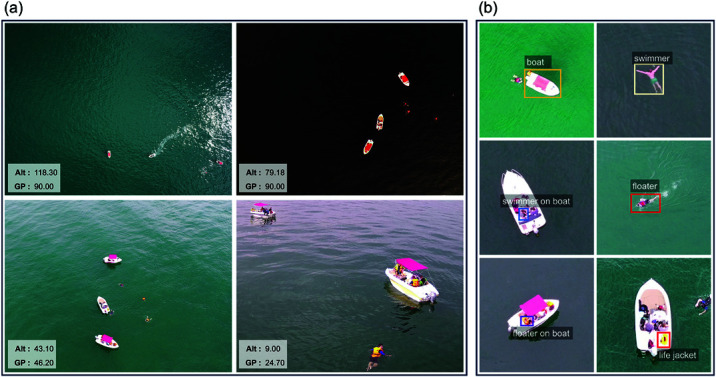
The SeaDronesSee dataset contains real-world drone-captured images of maritime search and rescue scenarios, presenting unique challenges for object detection. (a) Objects exhibit significant size and visual feature variations due to changes in drone flight altitude (Alt) and gimbal angle (GP), with substantial fluctuations in water surface patterns. (b) Close-ups of the scene show numerous small-sized targets with large size variations across different object categories, and overlap between boats, personnel, and equipment.

The high mobility of drones and their wide aerial coverage make drone-captured imagery a versatile tool across various fields, including traffic management, security surveillance, precision agriculture, and search and rescue operations [1–5]. Among these diverse applications, drone-based maritime search and rescue object detection stands out as a particularly valuable use case. By utilizing advanced object detection techniques to accurately identify and locate critical objects—such as rescue vessels, personnel, and lifesaving supplies—in drone-captured images over the ocean, drones can provide essential decision-making support for maritime rescue missions [6,7]. To facilitate research in this interdisciplinary domain, the large-scale SeaDronesSee dataset has been developed and made publicly accessible. As illustrated in Fig 1, this dataset captures the unique characteristics of real-world drone-based maritime search and rescue scenarios, offering a crucial foundation for advancing related studies.

The unique visual characteristics and task-specific challenges of drone-based maritime search and rescue scenarios, as presented in the SeaDronesSee dataset, have drawn considerable attention from the academic community [8–15]. Current research leveraging the SeaDronesSee dataset for object detection in this domain predominantly focuses on YOLO-based methods [16,17]. In comparison to traditional two-stage object detection models, such as Faster R-CNN and Cascade R-CNN, single-stage YOLO models eliminate the region proposal stage, treating object detection as a unified regression task [20,21]. This architectural simplicity inherently balances accuracy and computational efficiency, making YOLO models particularly well-suited for drone-based maritime search and rescue applications. The widespread adoption of YOLO in this research domain is driven by the dual requirements of high accuracy and lightweight design. These models must not only deliver precise detection results but also operate efficiently on resource-constrained drone platforms, where hardware limitations necessitate computationally efficient solutions [22,23].

While existing research utilizing the SeaDronesSee dataset for drone-based maritime search and rescue object detection has achieved commendable progress, notable research gaps remain. Most notably, Ultralytics recently introduced YOLO11 [24,25], the latest iteration in the YOLO series. Compared to its predecessors, YOLO11 incorporates substantial architectural innovations, including the C3k2 (Cross Stage Partial with kernel size 2) block and the C2PSA (Convolutional block with Parallel Spatial Attention) module. These advancements significantly enhance both feature extraction and computational efficiency. As a result, YOLO11 is particularly well-suited for maritime search and rescue applications, where achieving a balance between high accuracy and a lightweight design is critical to meet the computational limitations of drone platforms. To date, there has been no documented application of YOLO11 to the SeaDronesSee dataset for drone-based maritime search and rescue object detection. Optimizing YOLO11 for this specific domain presents an opportunity for significant advancements. Moreover, the official SeaDronesSee leaderboard places a strong emphasis on accuracy, which has driven current YOLO-based studies to prioritize precision [8,15], often at the expense of lightweight design—a critical consideration given the computational constraints of drone platforms. In response to these gaps, this study aims to refine the recently released YOLO11 model to enhance its performance in drone-based maritime search and rescue scenarios represented by the SeaDronesSee dataset. The proposed approach strives to achieve an optimal balance between accuracy and efficiency, addressing the dual demands of high performance and practical deployability in resource-constrained environments.

To achieve the research objectives, we conducted a thorough analysis of the image and sample characteristics within the large-scale SeaDronesSee dataset, which informed the formulation of specific research questions. Building on these insights, targeted optimizations were designed for the YOLO11 model to enhance its performance in drone-based maritime search and rescue object detection tasks. The effectiveness of the proposed approach was evaluated through extensive ablation studies and comprehensive comparisons against state-of-the-art methods. The experimental results provided critical evidence to address the identified research questions, demonstrating the potential of the optimized YOLO11 model to advance performance in this challenging domain.

Our contributions are threefold: First, we introduce YOLO11 into the study of drone-based maritime search and rescue object detection. Building on the characteristics of this task-specific dataset, we propose targeted research questions and corresponding optimizations tailored to YOLO11. Second, we conduct extensive ablation studies and comparative experiments against state-of-the-art methods on the large-scale SeaDronesSee dataset. These experiments validate the effectiveness of our proposed approach and highlight its performance advantages. Finally, we analyze the experimental results to identify both the strengths and limitations of the proposed method. Additionally, we outline key challenges and promising future directions for advancing maritime search and rescue target detection, as reflected in the insights gained from the SeaDronesSee dataset.

## Related work

Convolutional Neural Networks (CNNs) serve as a cornerstone architecture in deep learning, excelling at feature extraction from images through convolutional operations and nonlinear transformations [26]. Over time, CNNs have undergone substantial advancements, evolving from early models like LeNet to more sophisticated architectures such as AlexNet, VGGNet, and GoogLeNet [27–30]. These developments have progressively enhanced network depth, computational efficiency, and feature extraction capabilities [31,32]. The introduction of ResNet [33] further revolutionized the field by addressing the challenges of training very deep networks, enabling the development of deeper architectures with superior performance. More recently, innovations inspired by Capsule Networks have incorporated dynamic routing mechanisms, empowering CNNs to more effectively capture hierarchical structures and the deformation characteristics of objects in images [34–36]. These versatile CNN architectures are widely adopted as backbones in complex computer vision models, where they extract image features that are subsequently utilized in a broad range of vision tasks, including image classification, object detection, image segmentation, image captioning, and visual question answering [20,37–40]. Among these tasks, object detection stands out as one of the most critical challenges in computer vision, requiring accurate classification of objects within an image and precise localization of their positions using bounding boxes [20].

Object detection methods can generally be classified into two main categories: two-stage and one-stage approaches [20]. Two-stage detectors, such as Faster R-CNN [18] and Cascade R-CNN [19], first generate candidate regions through a region proposal stage and subsequently refine these proposals in a regression stage, where both object classification and bounding box regression are performed to achieve precise detection. In contrast, one-stage detectors, represented by YOLO [16,24,41] and SSD [42], reformulate object detection as a single-step regression task, directly predicting object categories and bounding boxes without an explicit region proposal phase. By eliminating this intermediate step, one-stage detectors typically feature a more streamlined architecture and significantly faster inference speed compared to their two-stage counterparts. This advantage makes them particularly well-suited for applications with limited computational resources and stringent real-time processing requirements. A prominent example of such a scenario is drone-based object detection, where onboard computational capacity is constrained, and real-time performance is often critical for operational success [43,44].

To facilitate the training and evaluation of object detection models, numerous large-scale datasets have been developed, including PASCAL VOC [45], MS COCO [46], and ImageNet [47], which predominantly feature images from everyday life scenes. However, aerial imagery captured from satellites, aircraft, and drones differs significantly from conventional images, exhibiting distinct characteristics that pose unique challenges for object detection. In response to these challenges, an increasing number of specialized datasets have been introduced, including remote sensing datasets such as DOTA [48], DIOR [49], and xView [50], as well as drone-based object detection and tracking datasets like VisDrone [51]. Among these, the SeaDronesSee dataset [52] stands out as a large-scale, publicly available dataset specifically designed for drone-based maritime search and rescue object detection. It addresses the inherent complexities of aerial imagery in maritime environments, providing a valuable benchmark for advancing research in this domain.

The development and public availability of the SeaDronesSee dataset provide a valuable resource for research on drone-based maritime search and rescue. However, due to the emphasis on average precision in the official SeaDronesSee leaderboard rankings [53], existing YOLO-based studies on this dataset [8,15] have predominantly focused on accuracy while overlooking the importance of lightweight model design. This accuracy-centric approach deviates from the original design philosophy of the YOLO series, which aims to balance both efficiency and precision. On the other hand, the most recent addition to the YOLO series, YOLO11 [24,25], released just a few months ago, is renowned for its ability to achieve an optimal trade-off between lightweight design and accuracy. It has demonstrated outstanding performance on general object detection benchmarks such as COCO [46]. However, compared to conventional images, the unique challenges posed by the SeaDronesSee dataset necessitate specialized optimizations for YOLO11 to fully exploit its potential in this domain. To the best of our knowledge, no prior research has been conducted on optimizing YOLO11 specifically for this scenario.

To address this research gap, this study introduces targeted optimizations for YOLO11 by systematically analyzing the unique characteristics and challenges posed by the SeaDronesSee dataset. Based on these optimizations, extensive experiments are conducted to rigorously assess the effectiveness of the proposed approach.

## Method

### Data analysis and research questions

SeaDronesSee [52] is a large-scale, publicly available dataset specifically designed for drone-based maritime search and rescue object detection. This dataset comprises 5,630 high-quality RGB images captured by various types of drones operating in real-world maritime search and rescue scenarios, with resolutions ranging from 1230×933 to 5456×3632 pixels. The creation and public release of the SeaDronesSee dataset provide a valuable resource for advancing research in drone technology, maritime search and rescue, and computer vision. Furthermore, the dataset’s unique visual characteristics introduce distinct challenges for image object detection, such as detecting small, overlapping, and rarely occurring objects. These features present rich opportunities for further research and the development of more robust detection models.

As illustrated in Fig 1(a), the visual characteristics of the same object can vary significantly depending on the drone’s altitude and gimbal pitch angle. For instance, at an altitude of 118.30 meters with a gimbal pitch angle of 90 degrees, a boat appears as a small, compressed shape in the image. In contrast, at an altitude of 9 meters with a gimbal pitch angle of 24.7 degrees, the boat occupies a larger portion of the image and is viewed from a side angle. Furthermore, the size disparity between different objects within the same frame is considerable. Boats appear much larger compared to people or life-saving materials, presenting additional challenges for object detection models. Compounding these difficulties, the sea surface exhibits significant variations in color and texture under different lighting conditions, further increasing the complexity of detection tasks. These unique characteristics of drone-based maritime search and rescue scenarios introduce significant challenges for the design and optimization of object detection models, necessitating advanced strategies to address these intricacies effectively.

The SeaDronesSee dataset encompasses six object categories to be detected, as depicted in Fig 1(b): boat, swimmer, floater, swimmer on boat, floater on boat, and life jacket. Table 1 provides detailed statistics for the training set, including sample counts and the minimum, maximum, and average areas (in pixels) for each category. The visual characteristics illustrated in Fig 1 and the statistical data in Table 1 highlight that the “boat” category has the largest object size and the most abundant samples. In contrast, the “swimmer” and “floater” categories, representing individuals on the sea surface, are significantly smaller in size compared to boats. The “swimmer on boat” and “floater on boat” categories, which depict individuals on boats, are characterized by smaller average object sizes, fewer samples, and frequent overlap with the “boat” category, posing additional detection challenges. The “life jacket” category presents the greatest challenge for precise detection. This category not only has the smallest object sizes and the fewest samples but also frequently overlaps with the “boat” category, further complicating its detection. These challenges underline the importance of developing advanced detection strategies tailored to the unique characteristics of this dataset.

**Table 1:** Sample counts and area statistics for object categories in the SeaDronesSee dataset.

Category	Samples	Mean Area	Min Area	Max Area
Swimmer	2480	5368.39	110	65601
Floater	5963	6631.17	50	172788
Boat	7643	31416.82	165	351330
Swimmer on Boat	3501	4323.04	108	35530
Floater on Boat	1603	4361.91	100	36736
Life jacket	82	987.34	336	2052

Given the unique visual characteristics of the drone-based maritime search and rescue scenario described above, this study addresses the following research questions:

- **RQ 1:** Despite YOLO11’s demonstrated success on general object detection datasets, what targeted optimization strategies can be developed to significantly enhance its detection capabilities in the specific context of drone-based maritime search and rescue?
- **RQ 2:** Compared to existing state-of-the-art YOLO-based object detection methods, what are the key advantages offered by the optimized YOLO11 model in the domain of drone-based maritime search and rescue?
- **RQ 3:** In the drone-based maritime search and rescue scenario, which object categories pose the greatest challenges for detection and optimization, and thus merit special attention in model refinement?

**Fig 2:**
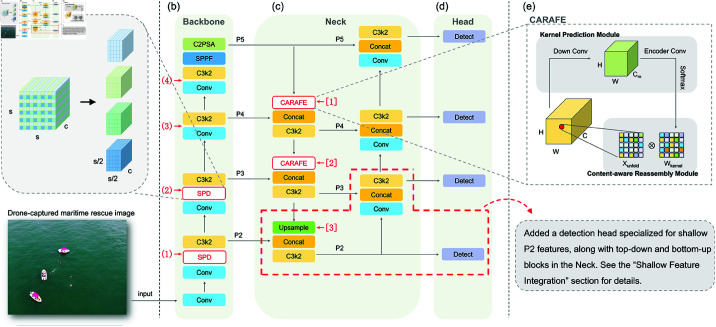
Overview of the proposed improvements to the YOLO11 model. (a) SPD illustration; (b) YOLO11 backbone with four potential SPD integration points (1)-(4), where (1) and (2) were selected based on ablation experiments; (c) YOLO11 neck with three potential CARAFE replacement points [1]–[3], where [1] and [2] were selected based on ablation experiments; (d) YOLO11 head, with red dashed lines indicating the additional detection head for P2 features and the corresponding neck modules; (e) CARAFE illustration.

To address the proposed research questions, we present a series of optimization strategies to enhance various components of the YOLO11 framework. The overall approach is illustrated in Fig 2. As shown in the figure, the Backbone of YOLO11 extracts multi-scale features (P2-P5) from shallow to deep layers, where P2 represents high-resolution shallow features suitable for detecting small objects, and P5 represents low-resolution deep features with rich semantic information for large objects.

To improve the model’s capacity for capturing fine-grained local visual features, we integrate a Space-to-Depth (SPD) operation [54] into the Backbone of YOLO11. This operation is designed to enhance the extraction of critical visual features, particularly for small and complex objects. Building on this foundation, additional modules are incorporated into the Neck, focusing on shallow P2 features, alongside an extra detection head in the Head section. These enhancements aim to improve the detection of small-scale objects, which are prevalent in drone-captured maritime search and rescue images. Furthermore, to achieve higher-quality feature reassembly and maximize the effective utilization of contextual information, we introduce the Content-Aware Reassembly of Features (CARAFE) upsampling algorithm [55] in the Neck section. This integration facilitates more robust feature aggregation, ultimately boosting the model’s overall detection performance in this challenging domain.

The following subsections provide a comprehensive discussion of the YOLO11-specific improvements and their adaptation to the unique characteristics of drone-based maritime search and rescue images. The proposed enhancements are carefully designed to address the challenges outlined in this domain. Subsequently, in the Experiment section, the effectiveness of these improvements will be rigorously evaluated through ablation studies and comparative experiments. These evaluations aim to validate the proposed approach while focusing on addressing the research questions formulated in this study.

### Space-to-depth enhanced backbone

As shown in Fig 2(a) and 2(b), the backbone of YOLO11 is responsible for extracting multi-level visual representations from drone-captured maritime search and rescue images, which are subsequently processed by the neck module for further refinement. To enhance the backbone’s feature extraction capability, we propose an optimized approach that incorporates the space-to-depth (SPD) module [54] into its architecture. The SPD module plays a crucial role by remapping the spatial information of input feature maps into the depth dimension. This transformation enables the network to capture and represent local contextual information more effectively, thereby improving its ability to detect small and complex objects commonly encountered in maritime search and rescue scenarios.

As illustrated in Fig 2(a), the workflow of the SPD module begins by reconstructing the input feature maps according to a spatial partitioning rule. Specifically, for a given factor *k*, the spatial resolution of the input feature map decreases from s×s to s/k×s/k, while the spatial information is redistributed into the depth dimension, increasing the feature map’s depth from *c* to $k^{2}\cdot c$.

In our design, the factor *k* is set to 2. The choice of *k* = 2 strikes a balance between performance gains and computational overhead, as validated in prior studies [54]. When *k* is increased to larger values, such as *k* = 4, the computational complexity rises significantly due to the exponential growth in the number of channels ($C^{\prime }=C\;\cdot \;k^{2}$), which compromises the lightweight design philosophy of YOLO11. Conversely, *k* = 2 effectively redistributes spatial information into the depth dimension, enhancing feature representation while maintaining computational efficiency. This careful trade-off ensures that the SPD module integrates seamlessly with YOLO11, preserving its real-time detection capabilities in maritime search and rescue scenarios.

As shown in Fig 2(a), each 2×2 block in the spatial domain is mapped into a single channel, thereby increasing the number of channels to four times the original value. Mathematically, given an input feature map *X* with dimensions [*C*,*H*,*W*], the SPD operation generates an output feature map $X^{\prime }$ with dimensions $\left[C^{\prime },H^{\prime },W^{\prime }\right]$, satisfying the following relationships:

$$C^{\prime }=C\cdot k^{2},\;H^{\prime }=\frac{H}{k},\;W^{\prime }=\frac{W}{k}.$$ (1)

Based on this principle, we incorporate the SPD module immediately before each C3k2 (Cross Stage Partial with kernel size 2) block in the YOLO11 backbone. This spatial transformation enhances the representational capacity of the feature maps, providing richer detail to the subsequent C3k2 blocks and later stages of the network. Integrating the SPD module into the YOLO11 backbone is expected to improve the model’s ability to capture fine-grained spatial features, which is particularly beneficial for drone-based maritime search and rescue tasks. By enhancing the feature maps at various stages of the network, the SPD module can help the model better detect small objects or those with overlapping positions, a common challenge in drone-based maritime object detection.

A critical question that arises from this design is whether integrating the SPD module at every possible position is necessary. As illustrated in Fig 2(b), there are four potential locations, labeled as (1)–(4), within the YOLO11 backbone where the SPD module can be inserted. However, incorporating the SPD module introduces additional computational complexity and increases the network’s overall computational cost. In the context of maritime search and rescue image-based object detection, determining the optimal locations for SPD integration requires empirical validation. The details of this investigation are addressed in the Experiment section, where we present a thorough ablation study to assess the trade-off between computational overhead and detection performance for each integration position.

### Shallow feature integration

As depicted in Fig 2(c) and 2(d), the default YOLO11 structure omits the components highlighted by red dashed lines. Notably, the P2 features extracted by the backbone, which are crucial for capturing fine-grained details and small objects, are not directly utilized by the neck or head components. This default design prioritizes a lightweight and computationally efficient implementation, which significantly reduces computational overhead while maintaining strong performance on general-purpose datasets, such as COCO [46] and VOC [45].

In the context of drone-based maritime search and rescue object detection, increasing drone altitude often leads to a proliferation of extremely small targets that require accurate detection. To address this challenge, this study enhances the YOLO11 architecture by incorporating additional neck and head components, as highlighted by the red dashed lines in Fig 2(c) and 2(d). These newly introduced components explicitly utilize the P2 features from the backbone, which are rich in local details and particularly well-suited for detecting small-scale objects. By leveraging these features, the enhanced architecture significantly improves the model’s ability to detect small targets in high-altitude drone imagery.

Specifically, the added components include two blocks in the neck’s top-down and bottom-up paths, as shown in Fig 2(c), and an additional detection head dedicated to P2 features, as shown in Fig 2(d). In the top-down path of the neck, the new block consists of three submodules: upsample, concat, and C3k2. This block not only receives the fused P3–P5 features from the previous top-down block but also directly connects to the C3k2 module responsible for P2 features in the backbone. The processed features from this block are then forwarded to the bottom-up path of the neck and the newly added detection head, which is dedicated to processing P2 features.

Following this is the new block in the bottom-up path, consisting of conv, concat, and C3k2 submodules. This block completes the neck’s top-down and bottom-up pathways, ensuring efficient multi-scale feature fusion.

The addition of these top-down and bottom-up blocks and the new detection head is expected to enhance the detection of small-scale objects in drone-based maritime search and rescue scenarios. However, these modifications significantly increase the model’s complexity and computational overhead. Therefore, the effectiveness of this strategy must be validated through ablation studies and comprehensive comparative experiments, as detailed in the Experiment section.

### CARAFE-driven multi-scale fusion

The neck of YOLO11 receives multi-level image features extracted from the backbone and fuses them through multi-scale feature aggregation, which are subsequently forwarded to the head for generating detection results. As illustrated in Fig 2(c), the YOLO11 neck leverages the upsample module, marked as [1]–[3] in the figure, to perform feature upsampling during the multi-scale fusion process.

While the default YOLO11 upsample module is sufficient for most image feature reconstruction tasks, it may fall short in addressing the unique challenges posed by object detection in drone-based maritime search and rescue images. In this specific application, adopting a higher-quality feature reassembly method is expected to more effectively utilize contextual information, thereby improving the quality of the upsampled features. This enhancement aims to make the processed features more expressive and capable of supporting downstream tasks.

Motivated by this observation, this study proposes replacing the default upsample module in YOLO11 with the Content-Aware Reassembly of Features (CARAFE) [55] module to enhance the neck’s ability to fuse multi-scale features. The CARAFE module’s content-aware reassembly mechanism is expected to significantly improve the detection capabilities of the modified YOLO11 model, particularly for targets of varying scales in drone-based maritime search and rescue images.

The core idea of CARAFE is to enhance the quality of upsampled features through content-aware reassembly, thereby supporting multi-scale fusion more effectively. As shown in Fig 2(e), CARAFE consists of two key components: the Kernel Prediction Module and the Content-Aware Reassembly Module, which work complementarily in the upsampling process. The Kernel Prediction Module generates adaptive upsampling kernel weights based on the input feature map. Given an input feature map $X\in {\mathbb{R}}^{C\times H\times W}$, the kernel weights are computed as:

$$W_{\text{kernel}}=\text{softmax}(\text{Conv2D}(\text{Conv2D}(X))),$$ (2)

where $W_{\text{kernel}}\in {\mathbb{R}}^{(K^{2}\cdot S^{2})\times H\times W}$ represents the adaptive convolution kernel weights for each spatial position.

The Content-Aware Reassembly Module dynamically reassembles the input feature map using the kernel weights generated by the Kernel Prediction Module. Specifically, the reassembled output feature *Y* is computed as:

$$Y_{\left(n,c,h^{\prime },w^{\prime }\right)}=\sum_{k=1}^{K^{2}}X_{\text{unfold}}(n,c,k,h,w)\cdot W_{\text{kernel}}(n,k,h,w),$$ (3)

where $h^{\prime },w^{\prime }$ denote the upsampled spatial positions, and $X_{\text{unfold}}$ represents the local receptive fields extracted from the input feature map *X*. Finally, the upsampled output is obtained by applying a pixel shuffle operation. This process achieves high-quality, content-aware feature upsampling, providing high-fidelity feature representations for multi-scale object detection tasks in drone-based maritime search and rescue images.

It is worth noting that, as shown in Fig 2(c), the neck of YOLO11 contains three upsample modules, marked as [1]–[3] in the figure, that can be replaced by CARAFE. Although replacing all upsample modules theoretically improves the overall upsampling quality, such a global replacement may introduce additional computational overhead and does not necessarily provide equivalent performance gains at every position. Furthermore, the hierarchical design of YOLO11’s neck for multi-scale feature fusion suggests that the contributions of different upsample modules to feature representation may vary. Therefore, determining which upsample modules to replace with CARAFE becomes a critical issue in optimizing the network.

To address this, we conducted ablation study to evaluate the impact of different replacement configurations on the model’s performance in the context of drone-based maritime search and rescue object detection. These experiments enabled us to identify the optimal configuration for incorporating CARAFE into the neck. Details of the experimental setup and results are provided in the Experiment section.

## Experiment

### Experimental setup and evaluation metrics

To validate the effectiveness of the proposed method, various drone-based maritime search and rescue object detection models were trained and evaluated on an Ubuntu workstation equipped with an RTX 3090 GPU and an Intel 14600KF CPU. The models’ accuracy and computational complexity were systematically compared to assess their performance. For training and evaluation, the SeaDronesSee dataset [52], a large-scale, publicly available dataset discussed in the Method section, was utilized. The official training set, comprising 2,975 images, was used to train the models. Their performance was subsequently evaluated on the validation set, consisting of 859 images, to measure object detection accuracy across various categories.

All models were trained for 300 epochs using a batch size of 16 and an image size of 640. The YOLOv8, YOLO11, and the proposed optimized YOLO11 models were trained from scratch, with hyperparameters set to the default values provided by Ultralytics [24]. For MambaYOLO [56] and YoloOW [15], the training configurations followed the settings described in their respective original papers and GitHub repositories [57,58]. To ensure a fair comparison, the image size for YoloOW was adjusted from its official setting of 1280 to 640 to align with the settings used for the other models. All other hyperparameters and pre-trained weights for YoloOW were kept consistent with its official configuration, ensuring that the evaluation accurately reflected the performance of each model under comparable conditions.

We adopt *AP*_50−95_ as the evaluation metric to assess the object detection performance of various models in drone-based maritime search and rescue scenarios. *AP*_50−95_ represents the average precision of a model across multiple IoU thresholds. Specifically, *AP*_50−95_ is computed by averaging the precision values at IoU thresholds ranging from 0.50 to 0.95 in increments of 0.05. Mathematically, this can be expressed as:

$$AP_{50-95}=\frac{1}{10}\sum_{i=50}^{95}AP(i)$$ (4)

where *AP*(*i*) denotes the average precision at an IoU threshold of *i*/100. This metric provides a comprehensive evaluation of model performance across varying detection precision requirements. It is particularly valuable for tasks involving diverse target sizes and complexities, such as those encountered in maritime search and rescue scenarios. For brevity, we refer to *AP*_50−95_ as AP in the subsequent sections of this paper.

Compared to single-point metrics like precision or recall, *AP*_50−95_ provides a more comprehensive evaluation of a model’s detection performance across different IoU thresholds. This makes it particularly suitable for scenarios like UAV-based maritime search and rescue, where targets vary significantly in size, appearance, and complexity. In our experiments, *AP*_50−95_ are calculated for all six object categories individually, as well as their overall average, ensuring a detailed and reliable evaluation of model performance across different object classes. Furthermore, as this study focuses on evaluating the trade-off between model accuracy and complexity, *AP*_50−95_ is the most representative metric for this purpose. It has been widely adopted in state-of-the-art object detection research [24,58], due to its ability to reflect the overall detection performance across multiple IoU thresholds.

To evaluate model complexity, we assessed two key metrics: the number of parameters and the number of floating-point operations (FLOPs). The number of parameters is expressed in millions, denoted as Params (M), while FLOPs are measured in billions, denoted as FLOPs (G). Params (M) quantifies the total number of trainable parameters in a model, providing an indication of its size and storage requirements. In contrast, FLOPs (G) measures the number of floating-point operations performed during a single forward pass, reflecting the computational cost of inference. Higher FLOPs values correspond to increased computational demands and greater hardware resource requirements. Together with AP_50−95_, these metrics offer a comprehensive evaluation framework for comparing both the performance and complexity of the models in our experiments. By examining these metrics in conjunction, we provide a balanced assessment of the trade-offs between accuracy, computational efficiency, and model size.

### Ablation study

We propose three targeted optimization strategies for the YOLO11 model to enhance its performance in drone-based maritime search and rescue object detection. These strategies, discussed in detail within the subsections of the Method section, include: Shallow Feature Integration (SFI), the incorporation of Space-to-Depth (SPD) modules into the backbone, and the integration of CARAFE upsampling in the neck. To evaluate the effectiveness of these strategies, we designed and conducted ablation experiments on the “s” scale YOLO11 model—a lightweight variant with both network depth and width scaled to 0.5—specifically tailored to meet the lightweight requirements of drone-based applications. These enhancements were progressively integrated into the original YOLO11 architecture.

The ablation experiments are designed with two primary objectives. First, we progressively integrate the three optimization strategies proposed in the Method section—SFI, SPD, and CARAFE—into the original YOLO11 framework, observing changes in AP to verify the effectiveness of each strategy. Second, since the SPD and CARAFE modules can be incorporated at multiple positions within the architecture, as indicated in Fig 2, specifically positions (1)–(4) for SPD and [1]–[3] for CARAFE, we conduct ablation experiments to identify the optimal positions for integrating these modules.

As shown in Fig 2, incorporating the SFI strategy into the YOLO11 framework impacts the integration of SPD and CARAFE in positions (1) and [3], respectively. Therefore, SFI was first integrated into the YOLO11 framework to comprehensively evaluate the effects of introducing SPD and CARAFE at the positions marked as (1)–(4) and [1]–[3] in subsequent steps. As indicated by the first two rows in Table 2, integrating SFI into YOLO11 improved the mean average precision by 21.43% on the SeaDronesSee dataset. Compared to the default YOLO11 architecture, the SFI-enhanced model, which includes a detection head and neck specifically designed for shallow features, showed improved detection accuracy across all categories except for “life jacket.” Notably, significant improvements were observed in detecting small overlapping objects, particularly “swimmer on boat” and “floater on boat.” Rows 3 to 6 in Table 2 present the ablation study results for the SPD strategy. Building upon the integration of the SFI strategy, SPD modules were progressively added to positions (1) through (4) as marked in Fig 2(b), evaluating their impact on model performance and computational cost. The results indicate that while the computational overhead, measured in GFLOPs, increased with each additional SPD module, the corresponding improvements in mAP did not scale proportionally. Specifically, the SPD12 model, which integrates SPD modules only at positions (1) and (2), achieved a better balance between mAP and GFLOPs. Notably, SPD12 attained the highest precision among the four SPD configurations for the “life jacket” category, which is the most challenging class to detect.

**Table 2:** Ablation study results.

Model	SFI	SPD	CARAFE	S	F	B	S*	F*	LJ	mAP	FLOPs
YOLO11	×	×	×	32.1	32.0	73.5	4.8	0.4	0.0	23.8	21.3 G
SFI		×	×	34.4	39.0	74.3	14.1	11.8	0.0	28.9	34.5 G
SPD1		(1)	×	34.1	38.9	75.8	14.3	14.7	0.0	29.6	38.6 G
SPD12		(1)(2)	×	35.0	37.9	70.2	9.8	10.9	19.8	30.6	44.9 G
SPD123		(1)(2)(3)	×	34.9	38.2	72.7	11.7	12.9	14.9	30.9	51.6 G
SPD1234		(1)(2)(3)(4)	×	36.1	39.3	73.4	14.3	11.8	9.9	30.8	55.0 G
CARAFE1		(1)(2)	[1]	**37.7**	41.0	73.5	**15.0**	12.6	0.0	30.0	45.6 G
CARAFE12		(1)(2)	[1][2]	36.1	**41.4**	74.7	13.1	**15.0**	**24.8**	**34.2**	45.5 G
CARAFE23		(1)(2)	[2][3]	37.7	41.0	73.5	15.0	12.6	0.0	30.0	45.6 G
CARAFE123		(1)(2)	[1][2][3]	35.0	41.1	75.4	14.5	13.7	0.0	29.9	45.9 G

*Table notes:* The table presents the ablation study results for the three optimization strategies proposed in the Method section. The ✓ and × in the SFI column indicate whether the SFI strategy is integrated. The SPD and CARAFE columns use (1)–(4) and [1]–[3] to indicate the positions where SPD modules were added and CARAFE modules replaced upsample modules, as shown in Fig 2. Columns S, F, B, S*, F*, and LJ represent the ${\text{AP}}_{50\text{-}95}$ scores for the categories “swimmer,” “floater,” “boat,” “swimmer on boat,” “floater on boat,” and “life jacket,” respectively, while the mAP column represents the average ${\text{AP}}_{50\text{-}95}$ score across all categories.

Consequently, in the subsequent ablation studies on CARAFE, shown in the last four rows of Table 2, the SPD12 configuration was chosen as the baseline, retaining SPD modules only at positions (1) and (2). The CARAFE modules were then further evaluated to determine their optimal integration.

As shown in the table, the ablation study results for CARAFE reveal that this optimization strategy, which replaces rather than adds to the existing upsample module, does not significantly alter the computational cost measured in FLOPs. In terms of model precision, replacing the upsample module with CARAFE at positions [2] and [3], or at position [1] alone, as indicated in Fig 2(c), offers slight advantages in detecting “swimmer” and “swimmer on boat” objects. However, the model referred to as CARAFE12 in the table, which incorporates CARAFE at positions [1] and [2], demonstrates superior performance across most object categories, achieving the highest average precision among all configurations. Notably, CARAFE12 excels in detecting the “life jacket” category, the most challenging object to identify, making it the most effective model in terms of overall precision in this ablation study.

In summary, the ablation study results demonstrate that progressively integrating the three optimization strategies proposed in the Method section into the “s” scale YOLO11 framework effectively enhances the model’s detection accuracy for drone-based maritime search and rescue tasks. Specifically, as shown in Table 2, the YOLO11 model, incrementally incorporating the SFI, SPD12, and CARAFE12 strategies, achieves notable improvements in mAP compared to the default YOLO11 architecture. These enhancements translate to relative mAP increases of 21.43%, 28.57%, and 43.70%, respectively, on the SeaDronesSee dataset, highlighting the effectiveness of these optimizations for this challenging task. The best-performing model in the ablation study, YOLO11-SFI-SPD12-CARAFE12, represents the proposed method and will be comprehensively compared with YOLO-based state-of-the-art methods in the next subsection.

### Comparison with state-of-the-art methods

In order to further validate the effectiveness of the proposed YOLO11 optimization for real-time object detection in UAV-based marine search and rescue scenarios, a comprehensive comparison is conducted between our method and four other state-of-the-art YOLO-based models. Table 3 presents a detailed comparison of detection performance across various object categories and computational overhead on the SeaDronesSee dataset, considering model complexities ranging from lightweight to heavyweight.

**Table 3:** Comparison with YOLO-based state-of-the-art methods.

Model	S	F	B	S*	F*	LJ	mAP	Params	FLOPs
YOLOv8-n [24]	29.8	32.2	72.0	3.3	0.1	0.0	22.9	3.0 M	8.1 G
YOLOv8-s [24]	34.7	34.1	72.9	3.0	0.4	0.0	24.2	11.1 M	28.5 G
YOLOv8-m [24]	36.9	35.4	73.0	6.5	4.5	0.0	26.1	25.8 M	78.7 G
YOLOv8-l [24]	34.4	35.3	73.7	6.0	6.0	0.0	25.9	43.6 M	164.8 G
YOLOv8-x [24]	35.4	35.7	73.9	6.8	5.1	0.0	26.1	68.1 M	257.4 G
YOLO11-n [24]	28.4	29.9	73.7	3.3	0.2	0.0	22.6	2.6 M	6.3 G
YOLO11-s [24]	32.1	32.0	73.5	4.8	0.4	0.0	23.8	9.4 M	21.3 G
YOLO11-m [24]	36.6	34.9	74.8	7.2	4.5	0.0	26.3	20.0 M	67.7 G
YOLO11-l [24]	36.4	36.5	73.0	5.3	3.6	0.0	25.8	25.3 M	86.6 G
YOLO11-x [24]	36.5	35.8	74.6	6.5	7.8	0.0	26.9	56.8 M	194.4 G
MambaYOLO-T [56]	29.7	31.0	73.1	3.3	0.0	0.0	22.9	6.0 M	13.6 G
MambaYOLO-B [56]	32.8	33.8	73.8	3.6	2.3	0.0	24.4	21.8 M	49.6 G
MambaYOLO-L [56]	36.4	35.9	74.3	5.7	0.7	0.0	25.5	57.6 M	155.9 G
YoloOW-n [15]	29.5	28.4	64.3	1.6	3.7	0.0	21.2	2.7 M	6.1 G
YoloOW-s [15]	33.5	33.7	68.2	5.8	7.8	0.0	24.8	10.6 M	24.0 G
YoloOW-m [15]	34.8	36.5	70.9	11.3	16.2	0.0	28.3	23.7 M	53.5 G
YoloOW-l [15]	36.3	39.5	74.7	18.0	24.4	24.9	36.3	42.1 M	94.8 G
YoloOW-x [15]	34.8	38.4	72.4	15.6	16.9	5.0	30.5	94.6 M	212.6 G
ours-n	32.3	35.0	74.5	6.6	10.9	0.0	26.5	2.9 M	14.7 G
ours-s	36.1	41.4	74.7	13.1	15.0	24.8	34.2	10.7 M	45.5 G
ours-m	36.8	42.8	74.5	19.6	16.5	7.4	32.9	24.6 M	154.4 G
ours-l	36.7	43.5	73.9	16.6	18.1	9.9	33.1	30.8 M	187.0 G
ours-x	39.5	41.9	74.2	18.1	14.1	14.9	33.8	68.2 M	410.1 G

*Table notes:* This table provides a comprehensive comparison of our improved YOLO11-based models and other YOLO-based state-of-the-art models on the SeaDronesSee dataset. The suffixes n, s, m, l, x, T, B, and L denote variants with different complexity levels, determined by varying the network’s width and depth scale factors. Columns S, F, B, S*, F*, and LJ represent the AP scores for the categories “swimmer,” “floater,” “boat,” “swimmer on boat,” “floater on boat,” and “life jacket,” respectively, while the mAP column indicates the average ${\text{AP}}_{50\text{-}95}$ score across all categories.

Specifically, the model labeled as “ours” in the table adopts the optimization strategy which was shown to yield the best performance in the Ablation Study section. This strategy integrates the additional YOLO11 detection head and Neck module described in the “Shallow Feature Integration” subsection, along with SPD integrated at the positions marked as (1) and (2) in Fig 2, and CARAFE integrated at the positions marked as [1] and [2]. For comparison, the state-of-the-art methods considered in this study include YOLO11, YOLOv8, MambaYOLO, and YoloOW. Specifically, YOLO11 and YOLOv8 are the latest and previous official versions from Ultralytics [24], respectively. MambaYOLO [56], a recently proposed object detection method based on State Space Models (SSM), demonstrates a substantial performance improvement over the existing YOLO series on the COCO and VOC datasets. YoloOW [15], on the other hand, is a model specifically optimized for UAV-based marine search and rescue detection tasks. The proposed OaohRep convolutional module and UAV Detection Box Filter significantly enhance the YOLO model’s adaptability and accuracy in multi-scale object detection within this specialized scenario, and YoloOW has maintained the top spot on the SeaDronesSee official leaderboard [53] for accuracy over an extended period.

To comprehensively evaluate the performance of various models in drone-based maritime search and rescue scenarios presented by the SeaDronesSee dataset, we trained versions of each model with varying levels of complexity. Specifically, the YOLOv8, YOLO11, and our models, as well as YoloOW, were evaluated across scales from lightweight to complex, labeled as “n”, “s”, “m”, “l”, and “x”. These scales follow the configurations provided in the official Ultralytics code [24] for the corresponding YOLO versions. For MambaYOLO, the “T”, “B”, and “L” scales are defined according to the original paper and code repository [56,58], with depth and width scaling factors of (0.33, 0.25), (0.33, 0.50), and (0.67, 0.75), respectively.

**Fig 3:**
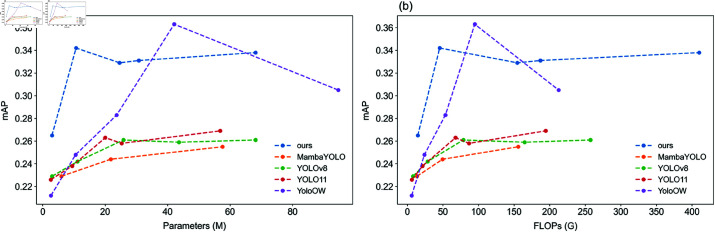
Performance comparison between the proposed method and YOLO-based state-of-the-art methods on the SeaDronesSee dataset under varying model complexities. (a) mAP curves for different parameter scales; (b) mAP curves for different computational complexities.

To provide a more intuitive comparison of model performance at varying levels of complexity, we present the line chart in Fig 3. As shown in both Table 3 and Fig 3, MambaYOLO performs the worst on the SeaDronesSee dataset. Although the SSM-based MambaYOLO outperforms official YOLO models on general datasets such as COCO and VOC by better capturing object features, the related optimizations do not translate well to the task of multi-scale small object detection for personnel and boats in UAV-based maritime search and rescue scenarios. Additionally, YOLOv8 and YOLO11 perform better than MambaYOLO in these scenarios, with YOLO11 slightly outperforming YOLOv8 overall, despite some performance fluctuations between the “m” and “l” scales. According to the data in the table, these fluctuations in YOLO11 and YOLOv8 are primarily due to the detection of small-size targets such as “swimmer on boat” and “floater on boat” , which overlap with boats. It is also noteworthy that all three models—MambaYOLO, YOLOv8, and YOLO11—achieved a precision of 0 for detecting the “life jacket” class, highlighting a major challenge for model optimization in accurately detecting this category.

As shown in the curve in Fig 3 and the data in Table 3, the YOLO11 model optimized by our proposed approach outperforms YOLOv8, YOLO11, and MambaYOLO across all levels of model complexity. Moreover, although our method exhibits lower average precision than YoloOW within a specific high-complexity and high-computational-cost range, it significantly outperforms YoloOW, which is known for its high precision in UAV maritime search and rescue detection, in the lightweight range (i.e., with fewer than 30 million parameters and below 70 GFLOPs).

According to the original paper of YoloOW [15], the recommended configuration (the “l” scale in Table 3) shows that YoloOW performs significantly better than two-stage detection models with complex backbones at the same level of computational complexity and cost. In other words, the optimization goal of YoloOW is to achieve high precision for UAV maritime search and rescue detection under higher computational cost. In contrast, our optimization goal is to achieve superior UAV maritime search and rescue detection within the lightweight range, using a more streamlined and low-cost YOLO11 model. The data from both the table and the figure clearly demonstrate that our proposed optimization successfully achieves this goal.

### Quantitative and qualitative analysis of YOLO11 baseline and optimized model

In the previous subsection, we validated the accuracy of the YOLO11 model integrated with the proposed optimization strategies under varying levels of model complexity. The experimental results demonstrated that, at the “s” scale, where both the depth and width of the model are reduced to 0.5 for lightweight implementation, the optimized model exhibits significant advantages in both efficiency and accuracy for drone-based maritime search and rescue scenarios. In this subsection, we further conduct a quantitative and qualitative comparative analysis between the YOLO11 baseline and our optimized model. This analysis aims to reveal the effectiveness of the proposed optimizations and provide insights into the underlying reasons for their performance improvements through both statistical evaluations and visualizations.

Table 4 summarizes the differences between the YOLO11 baseline and the YOLO11 model enhanced with the three optimization strategies proposed in this study under the “s” scale configuration. Columns 2 to 7 in the table illustrate the variations in detection performance for six object categories before and after optimization. Specifically, the improvement for the “boat” category is the most modest, with AP_50−95_ increasing from 73.5 to 74.7, representing a gain of only 1.63%. In contrast, significant improvements are observed for the detection of “swimmer” and “floater” on the water surface, with AP_50−95_ increasing by 12.46% and 29.36%, respectively, relative to their baseline values. Even more substantial gains are achieved for the categories “swimmer on boat” and “floater on boat,” where AP_50−95_ increases dramatically from 4.8 and 0.4 to 13.1 and 15.0, respectively. Notably, the “life jacket” category, which was completely undetected in the baseline model, reaches an AP_50−95_ of 24.8 in the optimized model.

**Table 4:** Comparison of the YOLO11 baseline and our optimized model at the “s” scale.

Model	S	F	B	S*	F*	LJ	mAP	Params	FLOPs	TPI
YOLO11-s [24]	32.1	32.0	73.5	4.8	0.4	0.0	23.8	9.4 M	21.3 G	2.5 ms
Ours-s	36.1	41.4	74.7	13.1	15.0	24.8	34.2	10.7 M	45.5 G	3.3 ms
Difference	(+4.0)	(+9.4)	(+1.2)	(+8.3)	(+14.6)	(+24.8)	(+10.4)	(+1.3 M)	(+24.2 G)	(+0.8 ms)

*Table notes:* The columns S, F, B, S*, F*, and LJ represent the ${\text{AP}}_{50\text{-}95}$ scores for the categories “swimmer,” “floater,” “boat,” “swimmer on boat,” “floater on boat,” and “life jacket,” respectively, while the mAP column represents the average ${\text{AP}}_{50\text{-}95}$ score across all categories. Params and FLOPs indicate the number of model parameters and the computational cost, respectively. TPI (Time per Image) represents the inference time per image.

Overall, the model’s average detection accuracy across all target categories (as shown in the mAP column) improved from 23.8 to 34.2. This accuracy improvement was accompanied by increases in the model’s parameter count and computational complexity, with Params increasing by 1.3M and FLOPs by 24.2G. Since Params and FLOPs are static metrics, we additionally measured the inference time per image (TPI) for both the baseline and optimized models. The TPI was calculated using the YOLO framework’s validation interface, testing all images in the SeaDronesSee validation set under the experimental environment described in this chapter. The average TPI values recorded from the logs were 2.5 ms and 3.3 ms for the baseline and optimized models, respectively. This represents a 32% increase in inference time, which resulted in a significant 43.70% improvement in the model’s overall detection accuracy.

In summary, the YOLO11 model, integrated with the three optimization strategies proposed in this study, achieves significant improvements in accuracy at the “s” scale, with only modest increases in model complexity. These improvements are particularly notable for small objects on boat, exemplified by “life jacket,” “swimmer on boat” and “floater on boat” , where substantial performance gains are observed. Moderate enhancements are seen for objects on the water surface, namely “swimmer” and “floater.” In contrast, the performance improvement for the largest object category “boat” is negligibly small.

**Fig 4:**
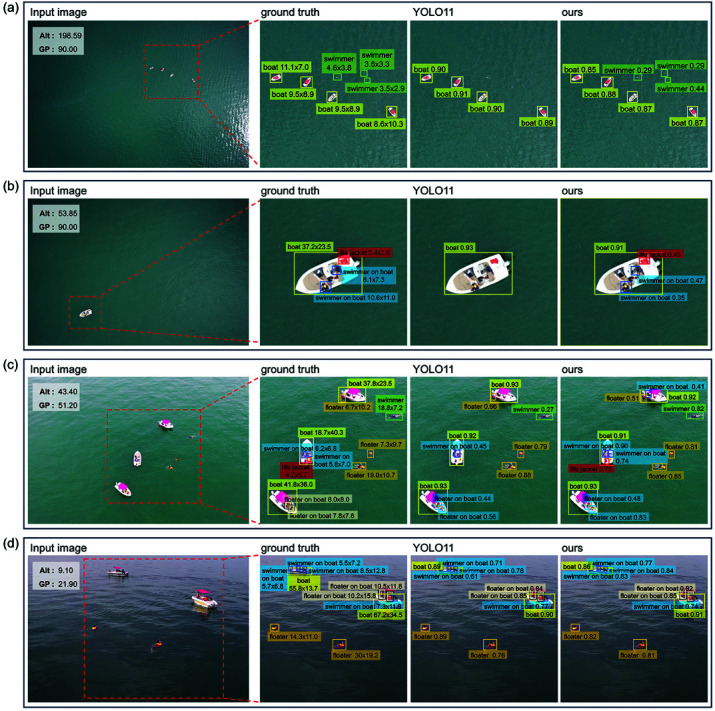
Ground-truth annotations and detection results visualization of representative images from the SeaDronesSee dataset captured at different altitudes.

To further observe the ability of the optimized model proposed in this paper compared to the YOLO11 baseline in detecting various object categories in drone-based maritime search and rescue scenarios, and to uncover the underlying reasons for its performance, the detection results are visually demonstrated in Fig 4. The figure illustrates representative images from the SeaDronesSee dataset, including ground truth annotations and the visualized detection results of the baseline and the optimized YOLO11 model before and after optimization.

Fig 4 comprises subplots (a)–(d), with image capture altitudes decreasing from 198.59 meters in (a) to 9.10 meters in (d). Each row consists of four images: the leftmost image shows the original input image provided to the model, while the three images to the right are zoomed-in details designed for visualization and analysis in the paper. These detailed views present the ground-truth annotations, detection results of the baseline YOLO11 model, and those of the optimized model, respectively. The ground-truth column visualizes the category labels of the objects present in the image, as well as their end resolution in the image, i.e., the height and width in pixels after preprocessing and resizing before inputting to the model. The detection results include bounding boxes, class labels, and confidence scores ranging from 0 to 1, where higher scores indicate stronger confidence in the detection results.

The image in Fig 4(a) was captured by a drone at an altitude of 198.59 meters with a 90-degree gimbal angle. Due to the high altitude, even “boat” objects appear as small shapes in the image, while “swimmer” objects on the water surface, with an end resolution ranging from 2.9 to 4.6 pixels, can only be observed as tiny forms in the magnified detail views. Objects on the boats are not visible and thus lack corresponding ground truth annotations. In detecting such high-altitude images, the YOLO11 baseline model can only identify “boat” objects, failing to effectively detect the tiny “swimmer” objects on the water. In contrast, our optimized model, leveraging the enhanced shallow feature utilization provided by SFI, the improved local contextual representation by SPD modules, and the high-quality feature reassembly enabled by CARAFE, achieves a confidence range of 0.29–0.44 in detecting “swimmer” objects. This demonstrates a significant improvement in the model’s ability to detect small targets in high-altitude scenarios.

At an altitude of 53.85 meters, as shown in Fig 4(b), objects on the boat, such as the “swimmer on boat” and “life jacket”, become visible and can be annotated as ground truth. These objects have an end resolution ranging from 3 to 11 pixels, but due to their overlap with the boat, they present additional challenges for object detection. The baseline YOLO11 model is only capable of detecting the boat itself, failing entirely to identify the smaller targets on the boat. In contrast, our optimized model, which integrates SFI, SPD, and CARAFE, effectively captures fine-grained features, enhances local contextual representations, and improves feature fusion quality. As a result, the optimized model achieves a confidence range of 0.35-0.47 when detecting these small and overlapping targets, demonstrating a significant improvement in detection performance, particularly in complex scenes.

Fig 4(c) presents a representative image captured at an altitude of approximately 40 meters. At this altitude, the boats, objects on the boats, and targets on the water surface are rendered with more detailed features. While the baseline YOLO11 model detects most object categories with moderate confidence, it fails to recognize the “life jacket” category due to its extremely small size and overlapping position with the boat. Our optimized model, however, leverages SFI to strengthen the utilization of shallow fine-grained features, while SPD, integrated at positions (1) and (2) as shown in Fig 2, enhances multi-scale representations of shallow and intermediate features, capturing contextual information critical for detecting small and overlapping objects. By avoiding integration at positions (3) and (4), which focus on higher-level features less relevant to this scenario, the model reduces computational overhead without sacrificing performance. Furthermore, the inclusion of CARAFE improves feature fusion quality, effectively balancing the retention of fine details with contextual reconstruction across shallow and intermediate features. This configuration strikes a balance between performance and efficiency, enabling the model to detect all object categories, including “life jacket”, with higher confidence. Notably, the optimized model successfully identifies a missed ground truth target, specifically “swimmer on boat 0.41” located in the upper region of the image, further validating the effectiveness of the proposed enhancements.

In the image shown in Fig 4(d), captured at a low altitude of 9.10 meters with a gimbal angle of 21.90 degrees, the details of boats, people, and objects are significantly clearer. The targets detected in this perspective closely resemble those typically encountered in everyday visual scenes. Consequently, both the baseline and optimized models are able to detect most targets in this low-altitude maritime search and rescue scenario with high confidence.

The analysis in this subsection demonstrates that the proposed improvements significantly enhance YOLO11’s performance in drone-based maritime search and rescue object detection, particularly in scenarios captured at high and medium altitudes. The SFI module strengthens shallow feature extraction, effectively improving the detection of small targets. The integration of SPD optimizes multi-scale contextual feature representation, notably enhancing the detection of small and overlapping targets in high- and medium-altitude scenes. Meanwhile, CARAFE’s high-quality feature fusion further improves the model’s adaptability to complex scenarios. Overall, the proposed optimization strategies address the specific feature requirements of different scenarios, achieving a balanced trade-off between performance and computational cost.

### Response to research questions

Based on the results of the ablation studies and comparisons with state-of-the-art methods, we provide the following answers to the research questions outlined in the Method section:

- **A1.** Targeted improvements to the YOLO11 architecture are both feasible and critically necessary to enhance its performance in the specific context of drone-based maritime search and rescue object detection. The proposed modifications to the backbone, neck, and head components effectively leverage shallow image features while facilitating better integration of multi-level features. These enhancements significantly improve the model’s capability to detect small and overlapping objects, which are prevalent in this scenario. Notably, within lightweight configurations—defined by FLOPs less than 50G and parameters under 11M—the optimized YOLO11 model achieves a relative mAP increase of 43.70% compared to the original YOLO11, highlighting the effectiveness of these targeted strategies.
- **A2.** The optimized YOLO11 model demonstrates substantial performance improvements over existing state-of-the-art YOLO-based models, including YOLOv8, YOLO11, and the recently proposed MambaYOLO, which excels on general-purpose datasets. These improvements are consistent across all complexity ranges in the domain of drone-based maritime search and rescue object detection. When compared to YoloOW, a model specifically optimized for this task, the proposed approach shows a significant advantage in the lightweight complexity range, defined by FLOPs less than 50G and Params less than 11M. Within this range, the optimized model achieves a 37.90% relative increase in mAP compared to YoloOW. This lightweight advantage is particularly valuable for real-world drone applications, where efficiency and suitability for deployment in resource-constrained and real-time object detection systems are critical.
- **A3.** The experimental results identify the “life jacket” category as the most challenging to detect within the SeaDronesSee dataset, with many models failing to achieve any detection accuracy for this category. Similarly, the “swimmer on boat” and “floater on boat” categories present significant detection challenges. As outlined in the Method section, these difficulties arise from the extremely small object sizes, frequent overlap with boats, and limited sample availability for these categories. Addressing these challenges is essential for future efforts in model optimization and dataset enrichment, as effective detection of these categories remains a critical objective for advancing performance in this domain.

## Discussion

This study integrates the recently released YOLO11 model into the task of drone-based maritime search and rescue object detection, as defined by the SeaDronesSee dataset, and proposes targeted optimizations tailored specifically to this application. The findings underscore the critical need to refine YOLO11 in order to address the unique challenges posed by this scenario. The optimized YOLO11 demonstrates substantial advantages over existing state-of-the-art methods, particularly in achieving higher accuracy within lightweight configurations.

Unlike approaches that prioritize accuracy at the expense of lightweight design, our method highlights the importance of balancing these two critical aspects. This balance not only aligns with the inherent strengths of the YOLO series but also addresses the practical constraints and demands of drone-based detection systems, where both efficiency and precision are indispensable.

Despite these contributions, this study has certain limitations that warrant discussion. First, the research focuses primarily on optimizing YOLO models to achieve high accuracy in drone-based maritime search and rescue object detection tasks under lightweight configurations. However, this focus does not explore the potential for achieving extreme accuracy through heavier configurations, computationally intensive methods, or meticulous fine-tuning. Such approaches could be particularly valuable in computation-rich environments, such as cloud-based drone systems. In this context, the methodologies adopted in YoloOW [15] serve as an excellent reference and are worth further exploration in future work.

Second, this study emphasizes algorithmic optimizations without addressing the deployment of the proposed model on real-world drone hardware systems or evaluating its generalization performance in practical scenarios. To address this limitation, future work could build upon existing approaches by exploring the deployment of the model within the edge-based paradigm, involving UAVs equipped with lightweight computing devices [59,60], in order to evaluate its performance under real-world conditions. Additionally, given the challenges associated with obtaining real-world drone maritime search and rescue data, future research could leverage virtual reality technologies to simulate drone maritime scenarios, as suggested by previous studies [14,61], to effectively mitigate this limitation. Furthermore, future research could explore the integration of sparse recovery methods from signal processing [67,68] with compressive sensing-based CNN compression techniques [69,70] in the context of real-world drone-based maritime search and rescue operations. Such integration could enhance system efficiency in handling target detection tasks in complex maritime environments, while simultaneously reducing the computational burden. This approach holds the potential to significantly improve both the real-time performance and detection accuracy of the YOLO11 model.

Third, the evaluation of model complexity in this study is based on static metrics such as Params, FLOPs, and runtime inference speed under experimental conditions. However, the model’s computational complexity and overhead remain to be validated and further optimized on real UAV platforms. A practical direction for future work is to deploy and evaluate the model on representative UAV hardware platforms, such as the NVIDIA Jetson series (e.g., Jetson Nano or Jetson Orin) [62,63], to quantify its inference speed, power consumption, and memory usage, followed by targeted adjustments and optimizations. On this basis, ensuring compatibility with edge computing devices in real-world scenarios, as well as optimizing real-time performance in dynamic maritime environments, also pose significant challenges. Strategies such as adaptive thresholding for detection confidence [64], integration with on-board sensors for environmental awareness [65], and selective activation [66] of the added components based on target size or scene complexity are promising directions to address these challenges effectively. Additionally, exploring the use of tools for generating heatmaps of feature activations could be a valuable direction for future work, offering qualitative insights into the model’s decision-making process, particularly for small or overlapping objects.

Finally, the experimental results reveal specific object categories within the SeaDronesSee dataset that pose significant detection challenges. Addressing these challenges by developing tailored optimization strategies for these hard-to-detect categories presents a promising direction for future research. Such efforts hold substantial potential to further enhance detection performance in this domain, particularly in scenarios where accurate identification of these objects is critical.

## Conclusion

This study introduces the recently released YOLO11 model to the task of drone-based maritime search and rescue object detection, as represented by the SeaDronesSee dataset, and proposes task-specific optimizations tailored to this unique application. Experimental results demonstrate that the optimized YOLO11 achieves significant performance improvements in this challenging scenario, surpassing existing state-of-the-art methods, particularly in delivering high accuracy under lightweight configurations. The findings of this research hold substantial promise for practical applications, including real-time detection and deployment on low-power devices for maritime rescue missions. Moreover, the proposed approach provides valuable insights into model optimization and serves as a useful reference for future studies in this domain. Future research could explore accuracy-focused model optimizations that prioritize performance over lightweight design, particularly for computation-rich environments. Furthermore, deploying and evaluating the proposed model in real-world drone systems to validate and enhance its generalization performance remains a crucial direction for future investigation.
